# Suanzao Ren Decoction Alleviates Anxiety‐Like Behavior and Cognitive Dysfunction in Chronic Unpredictable Stress Rats by Modulating Oxidative Stress and Autophagy

**DOI:** 10.1002/brb3.71585

**Published:** 2026-07-14

**Authors:** Tianyan Luo, Guifang Xiang, Qing Liu

**Affiliations:** ^1^ Department of Anesthesiology Hejiang Hospital of Traditional Chinese Medicine Luzhou Sichuan China; ^2^ Department of Painology Hejiang Hospital of Traditional Chinese Medicine Luzhou Sichuan China

**Keywords:** anxiety, autophagy, cognitive impairment, oxidative stress, Suanzao Ren Decoction

## Abstract

**Objective:**

This study investigated the effects and mechanisms of Suanzao Ren Decoction on anxiety symptoms of chronic unpredictable stress (CUS) model rats.

**Methods:**

A rat model of anxiety was established using chronic unpredictable stress. The open field test and elevated cross maze were used to evaluate the anxiety state of rats. The Morris water maze was used to evaluate the cognitive function of rats. HE staining was used to analyze the histopathological changes of the hippocampus. The protein expression of 5‐HT_2C_R, Parkin, and PINK1 in the hippocampus was detected by western blotting. Mitochondrial autophagy in the hippocampus was observed by transmission electron microscopy.

**Results:**

The treatment with Suanzao Ren Decoction significantly prolonged the total distance of spontaneous movement in rats, increased the percentage of open arm entry times, the percentage of open arm retention time, the retention time at the platform position, and the number of platform crossings, and shortened the escape latency period of rats. In addition, the treatment with Suan Zao Ren Decoction significantly improved the pathological damage in the CA1 area of the hippocampus, significantly increased the levels of SOD, CAT, GSH, 5‐HT, and TPH‐2, reduced the level of MDA, and significantly decreased the protein expressions of 5‐HT_2C_R, Parkin, and PINK1, as well as reduced the number of autophagic lysosomes.

**Conclusion:**

Suanzao Ren Decoction relieved anxiety behavior in CUS rats, and its mechanism may be related to improving cognitive dysfunction and inhibiting oxidative stress and autophagy.

## Graphical abstract

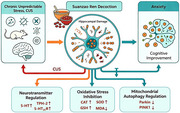

## 1 Introduction

Anxiety disorders, including generalized anxiety disorder, panic disorder, social anxiety disorder, etc., are the most common mental disorders, characterized by excessive worry, fear, and somatic symptoms, which severely affect the quality of life and social functioning of patients (Kowalchuk et al. 2022). In recent years, with the intensification of social competition and the acceleration of the pace of life, the incidence of anxiety disorders has been on the rise and has become an important public health issue worldwide (Xiang et al. 2024; Freeman 2022). The pathogenesis of anxiety disorders is complex and is related to abnormalities in neurotransmitters, neuroendocrine disorders, changes in neuroplasticity, genetic factors, and psychosocial factors (Freeman 2022). Oxidative stress, as an important pathophysiological mechanism, is closely related to the occurrence and development of anxiety disorders (Wu et al. 2022; Steyn 2025). Oxidative stress can lead to neuronal damage, neuroinflammation, and mitochondrial dysfunction, thereby affecting the normal function of the nervous system (Song et al. 2021; Picca et al. 2020). Inhibiting oxidative stress may become a target for improving anxiety symptoms.

Traditional Chinese medicine (TCM) herbs, as an important part of traditional Chinese medicine, have a long history and rich experience in the treatment of mental disorders (Tang et al. 2017). In recent years, with the in‐depth study of TCM herbs, more and more evidence has shown that TCM herbs have unique advantages in the treatment of anxiety disorders (Xie et al. 2024; Wang et al. 2023). TCM herbs have the characteristics of multiple components, multiple targets, and multiple pathways, and can exert anti‐anxiety effects through mechanisms such as regulating neurotransmitters, antioxidant stress, and anti‐inflammatory actions (Liu et al. 2025). In addition, TCM herbs have the advantages of fewer side effects and high safety, and have potential advantages in the long‐term treatment of anxiety disorders. TCM herbs may become a choice for the treatment of anxiety disorders (Trkulja and Barić 2020; Zhang et al. 2022). The first record of Suanzao Ren Decoction (Cheng et al. 2022) is in “Essential Prescriptions from the Golden Chamber,” which states, “For deficiency and restlessness with insomnia, Suanzao Ren Decoction is the principal formula,” and it is a classic prescription for treating deficiency and restlessness with insomnia and has significant anti‐anxiety and antidepressant effects. However, the mechanism of action of this compound formula is not yet clear.

This study, through multi‐level verification in animal experiments, revealed that in CUS rats, Suanzao Ren Decoction significantly improved anxiety‐like behavior, and indicated that Suanzao Ren Decoction exerts its anxiolytic effect by reducing oxidative stress, inhibiting excessive mitochondrial autophagy, and reducing neuroinflammation.

## 2 Materials and Methods

### 2.1 Animals

Thirty SPF‐grade male SD rats, aged 6–8 weeks and weighing 200–250 g, were purchased from Chengdu Dashuo Biotechnology Co., Ltd. All experimental procedures were approved by the Ethics Committee of the Affiliated Traditional Chinese Medicine Hospital of Southwest Medical University (No. swmu20220138) and strictly followed the Guide for the Care and Use of Laboratory Animals.

### 2.2 Drugs

Suanzao Ren Decoction consists of Suan zao Ren 15 g, gan cao 3 g, zhi mu 6 g, fu lin 6 g, and Chuan xiong 6 g and was purchased from the pharmacy of the Affiliated Traditional Chinese Medicine Hospital of Southwest Medical University.

### 2.3 Liquid Chromatography‐Mass Spectrometry/Mass Spectrometry (LC‐MS/MS)

The chemical composition of Suanzao Ren Decoction was analyzed and identified by LC‐MS/MS. In this experiment, a U3000 high‐performance liquid chromatograph (Thermo Fisher Scientific, USA) coupled with a Q Exactive mass spectrometer (Thermo Fisher Scientific, USA) was used for the separation and identification of the constituents contained in Suanzao Ren Decoction. Suanzao Ren Decoction (100 µL) was mixed with three times the volume of methanol and then vortexed and ultrasonicated for 30 min. It was then centrifuged at 12,000 rcf for 10 min at 4°C, and the supernatant was analyzed by LC‐MS.

Chromatographic conditions: ACQUITY Premier HSS T3 column (2.1 mm × 100 mm, 1.8 µm), mobile phase A was 0.1% formic acid, and mobile phase B was 100% methanol. The gradient elution conditions were as follows: 0–1 min, 2% B; 1–5.5 min, 2%–100% B; 5.5–14 min, 100% B; 14–14.1 min, 100%–2% B; and 14.1–16 min, 2% B. The column temperature was set at 45°C, the injection volume was set at 2 µL, and the flow rate was set at 0.3 mL/min.

Mass spectrometry conditions: electrospray ion source (ESI), positive and negative ion mode data acquisition. Data were processed using MS‐DIAL 4.70 software (RIKEN Tokyo, Japan).

### 2.4 CUS Model Preparation and Grouping

A chronic unpredictable stress (CUS) model of stress‐related anxiety‐like behavior was established in rats. Rats were randomly assigned one type of stressor daily for six weeks. The primary stressors included: 24 h light‐dark cycle reversal (lighting hours: 19:00–7:00; darkness hours: 7:00–19:00), complete water and food deprivation (19:00–7:00), 2 h restraint (restricting movement while maintaining free breathing and air circulation through fixation in restraints), foot‐pulse electric shocks (0.5 A current intensity, 5 min total, 5 s per min with 55 s intervals), forced swimming (10 min), bedding changes, and full‐day light exposure (7:00 PM to 7:00 AM the next day). Each stressor was applied five times on average, with different stressors not repeated within two consecutive days. The control group rats were fed with normal feed and water without any stimulation.

**FIGURE 1:**
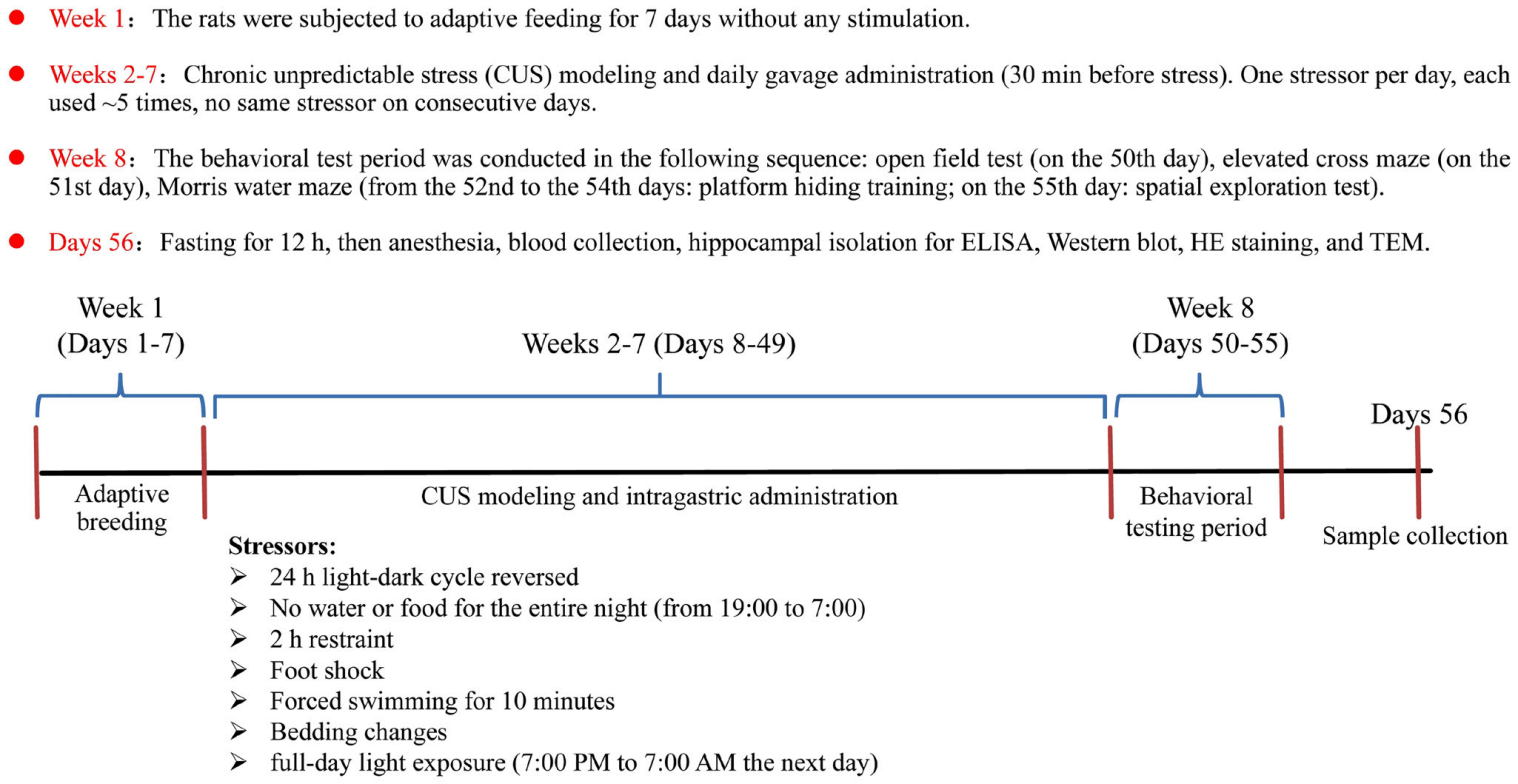
Experimental timeline.

Thirty rats were randomly divided into five groups: control group, model group, Suanzao Ren Decoction low‐dose group (SZRD‐L), Suanzao Ren Decoction high‐dose group (SZRD‐H), and fluoxetine group, with 6 rats in each group. Gavage administration was performed 30 min before daily stress exposure in rats. Based on equivalent human‐to‐rat body surface area dosing, the SZRD‐L and SZRD‐H groups received 6.48 g/kg and 12.96 g/kg of oral gavage, respectively, while the fluoxetine group received 0.0036 g/kg once daily for six consecutive weeks. The control and model groups received an equal volume of saline solution for six consecutive weeks. Behavioral experiments were conducted 24 h after drug termination. After completing behavioral experiments, the rats were fasted for 12 h. Following anesthesia with 1% pentobarbital sodium (50 mg/kg), hippocampal tissues were extracted and placed in 4% tissue fixative for preservation at 4°C. The detailed experimental timeline is shown in Figure 1.

### 2.5 Behavioral Experiments

Open field experiment (Wang et al. 2022): Rats were placed in the center of the bottom of the open field box, and the total travel of the rats in the open field box for 5 min was recorded by an automatic animal behavior tracking system. Keep the environment quiet during the test. After each experiment, wipe the entire open field chamber with 75% alcohol to avoid affecting the next experiment.

Elevated cross maze experiment (Maruyama 2025): A rat was placed in the center of the maze, with its head facing the open arm. At the same time, a camera monitor was turned on to record the number of times the rat entered the open arm, and the closed arm and the duration of their stay within 5 min.

Morris water maze experiment (Vörös et al. 2024): It includes the hidden platform test and the space exploration test. Hidden platform test: (1) The water maze is equally divided into four quadrants, with the center of the pool bottom as the center, representing quadrants I, II, III, and IV, respectively. The platform is placed in the target phenomenon (Quadrant I). The rats entered the water from each quadrant facing the pool wall and swam for 90 s to find the hidden platform. The escape incubation period is the time required to find a hidden platform in the pool. If the platform is still not found after 90 s, guide the rat to the platform, and the score will be counted as 90 s. The first four days are the training time. The rats with a score of 90 s were excluded, and the test score on the fifth day was recorded as the spatial learning and memory performance of the rats. (2) Space exploration test: 24 h after the hidden platform test is completed, remove the platform. Record the time the rats stayed in the *i*th quadrant and the number of times they crossed the platform within 60 s.

### 2.6 HE Staining

By referring to relevant materials (Cheng et al. 2022), hippocampal tissue samples were fixed in 4% paraformaldehyde for 48 h, followed by routine dehydration through graded ethanol series and clearing in xylene. The tissues were then embedded in paraffin and sectioned into 4 µm thick sections. After deparaffinization and rehydration, the sections were stained with hematoxylin for 5 min, rinsed in running tap water, differentiated in 1% hydrochloric acid alcohol for a few seconds, and then blued in Scott's tap water. Subsequently, the sections were stained with eosin for 3 min, dehydrated, cleared, and mounted with neutral resin. The histopathological changes in the hippocampus were observed under a light microscope (E100, Nikon, Japan).

### 2.7 ELISA

The hippocampal tissue was weighed and added to a cold PBS buffer for homogenization. The mixture was centrifuged at 3000 rpm, 4°C for 10 min. After taking the supernatant, the levels of superoxide dismutase (SOD), malondialdehyde (MDA), 5‐hydroxytryptamine (5‐HT), tryptophan hydroxylase 2 (TPH‐2), glutathione (GSH), and catalase (CAT) were detected according to the kit's instructions. SOD ELISA KIT (A001‐3‐2), MDA ELISA KIT (A003‐1‐2), 5‐HT ELISA KIT, CAT ELISA KIT (A007‐1‐1), and GSH ELISA KIT (A006‐2‐1) were purchased from Nanjing Jiancheng Bioengineering institute. TPH‐2 ELISA KIT (ml058852) was purchased from Shanghai Enzyme‐linked Biotechnology Co., Ltd.

### 2.8 Western Blotting

Hippocampal tissue samples were rapidly ground into homogenates at low temperatures. Protein was extracted using RIPA lysis buffer (Beyotime Biotechnology) and quantified with the Bradford Protein Concentration Kit. The extracted proteins were separated by SDS‐PAGE electrophoresis, transferred to polyvinylidene fluoride (PVDF) membranes, and blocked with 5% skim milk diluted in Tris buffer for 2 h. A 50 µL primary antibody was added and incubated overnight at 4°C. After PBS rinsing, a secondary antibody (goat anti‐rabbit IgG HRP) was added and incubated with the membrane at room temperature for 1 h. Finally, the target bands were developed using RapidStep ECL reagent. β‐actin was used as the internal control. Gray values were calculated using Image J software, and differential analysis was performed with SPSS statistical software.

### 2.9 Transmission Electron Microscope (TEM)

Hippocampal tissue was rapidly immersed in 3% glutaraldehyde for fixation for 2 h, followed by three PBS washes (15 min each). The tissue was then fixed in 1% osmium acid solution for 3 h, with three PBS washes (15 min each). Under 4°C conditions, the hippocampal tissue underwent sequential immersion in 50%,70%,90% ethanol, and a 90% ethanol–90% acetone mixture (1:1) for 30 min each, followed by 30 min in pure acetone at room temperature. The tissue was then placed in pure acetone with embedding medium (2:1) for 3 h, followed by overnight in pure acetone‐embedding medium (1:2), and finally 37°C incubation in pure embedding medium for 3 h. The tissue blocks were mounted on embedding slides and incubated overnight in a 45°C oven, then for 12 h in a 60°C oven. Semi‐thin sections were observed under light microscopy, with selected rat hippocampal tissue areas processed using an ultra‐thin sectioning machine to obtain 60–90 nm sections onto copper grids. The sections were stained with lead citrate for 10 min, washed three times with carbon dioxide‐free double‐distilled water, and stained with 3% acetic acid uranyl for 30 min, followed by three washes with carbon dioxide‐free double‐distilled water. Transmission electron microscopy images were captured from the copper grids.

### 2.10 Statistical Analysis

Statistical analysis was performed using SPSS 17.0 and GraphPad Prism 5. All data were presented as mean ± standard deviation. One‐way ANOVA was used for inter‐group comparisons. When variances were equal, the LSD‐*t* test was applied; when variances differed, Dunnett's *t*‐test was employed. A *p*‐value <0.05 was considered statistically significant.

## 3 Results

### 3.1 LC‐MS/MS Analysis of Suanzao Ren Decoction

**FIGURE 2:**
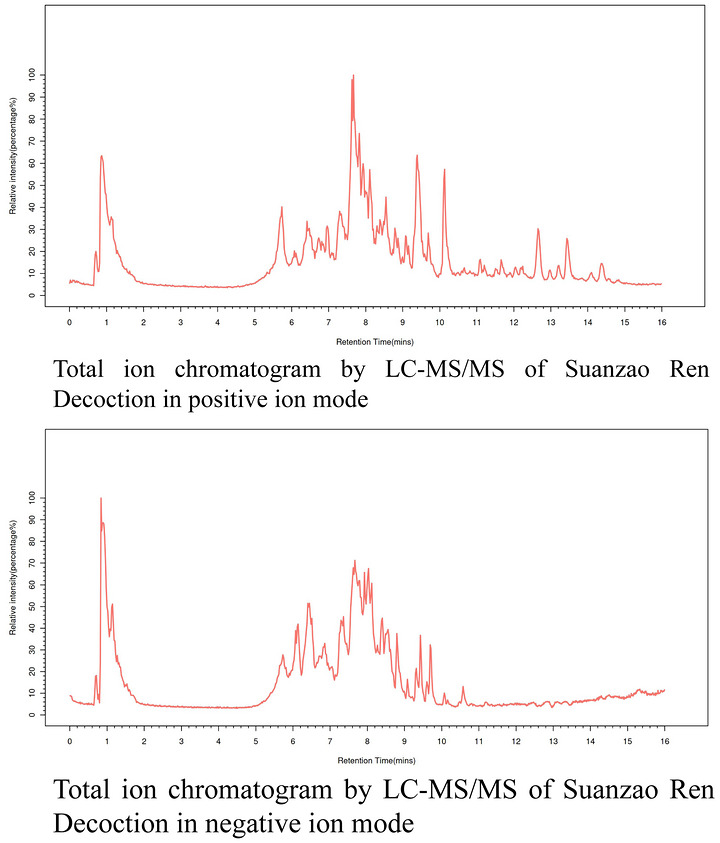
LC‐MS/MS analysis of Suanzao Ren Decoction. Total ion chromatogram in positive and negative ion modes of Suanzao Ren Decoction.

A total of 1271 active constituents were identified from Suanzao Ren Decoction by LC‐MS/MS analysis (Figure 2). The representative composition was “Mangiferin, N‐hydroxyl‐tryptamine, Carbenicillin Inulobiose, Justicidin b, Glycyrrhizin, and Isoliquiritigenin.”

### 3.2 Weight Changes in CUS Rats

**FIGURE 3:**
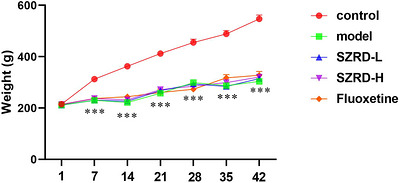
Weight changes in CUS rats. There were 6 rats in each group. ***p < 0.001 vs. control group.

The results (Figure 3) showed that compared with the control group, the other four groups of rats had significantly decreased body weight.

### 3.3 Effects of Suanzao Ren Decoction on Phenotype and Behavior of CUS Rats

**FIGURE 4:**
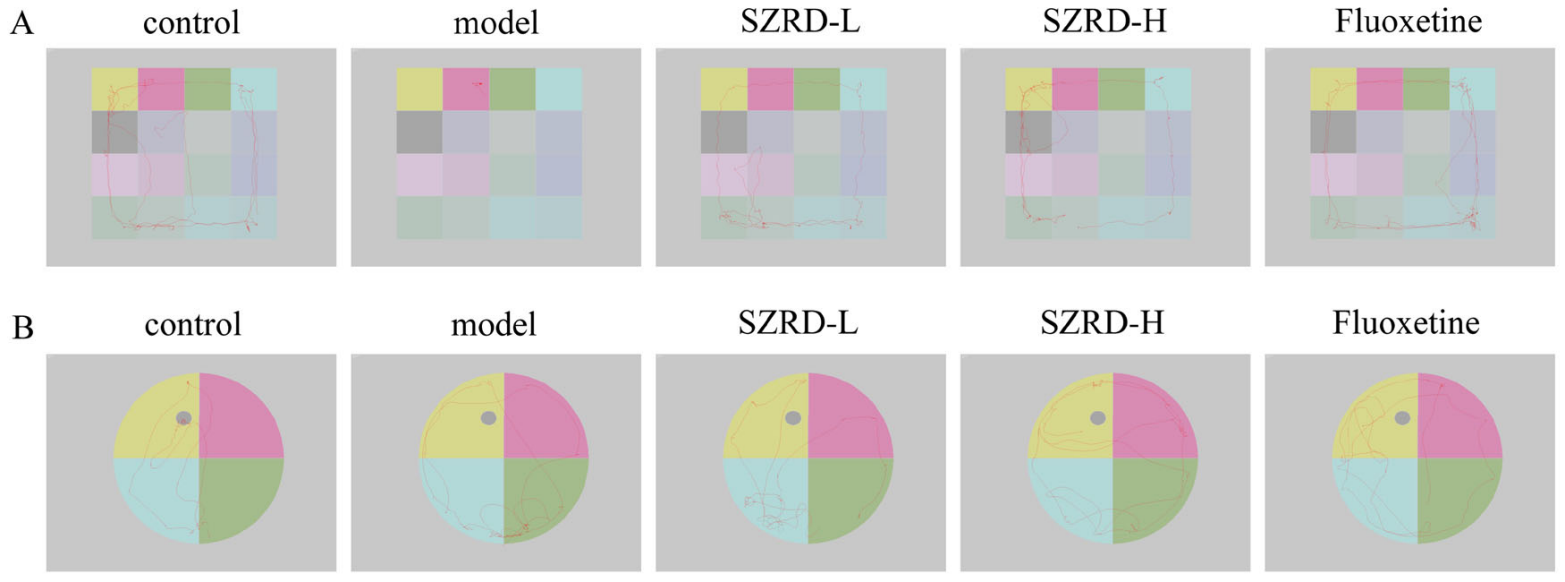
Trajectory map. (A) Open field test trajectory map. (B) Morris water maze trajectory map. There were 6 rats in each group. There were 6 rats in each group.

In the open field experiment (Table 1, Figure 4A), compared with the control group, the total distance of autonomous movement in model rats was significantly shortened. In the elevated cross maze (Table 1), the percentage of open arm entries and the percentage of time staying in the open arm decreased. In the water maze (Table 2, Figure 4B), compared with the control group, rats in the model group had a longer escape incubation period, a shorter platform stay time, and fewer times to cross the platform. After treatment with Suanzao Ren Decoction or fluoxetine, the total distance of autonomous movement in rats was significantly extended, the percentage of open arm entries, the percentage of time staying in the open arm, the duration of platform stay time and the number of times of crossing the platform were significantly increased, and the escape incubation period was shortened.

**TABLE 1:** **Open field test and elevated cross maze** $\left(\bar{x}\pm s\right)$.

Groups	Total distance of autonomous movement/cm	Percentages of open arm entries%	Percentage of time staying in the open arm%
Control	1208.68 ± 142.61	32.64 ± 5.74	34.34 ± 6.01
Model	232.52 ± 38.61***	8.12 ± 1.04***	9.31 ± 2.32***
SZRD‐L	536.98 ± 263.19	13.47 ± 1.92###	15.37 ± 3.16###
SZRD‐H	1014.42 ± 215.70###	16.08 ± 2.64###	18.25 ± 3.28###
Fluoxetine	1060.72 ± 163.80###	19.60 ± 2.85###	20.51 ± 3.39###

*Note*: There were 6 rats in each group. ***p < 0.001 vs. control group; ###*p* < 0.001 vs. model group.

**TABLE 2:** **Morris water maze test** $\left(\bar{x}\pm s\right)$.

Groups	Escape incubation period/s	Duration of platform stay time/s	Number of times of crossing the platform
Control	33.20 ± 3.11	26.8 ± 1.04	8.01 ± 0.49
Model	67.14 ± 5.45***	13.5 ± 1.13***	2.26 ± 0.58***
SZRD‐L	51.28 ± 8.25###	20.5 ± 1.12###	3.88 ± 0.97###
SZRD‐H	40.15 ± 3.29###	24.2 ± 0.88###	5.36 ± 0.19###
Fluoxetine	42.88 ± 6.37###	22.6 ± 0.79###	6.52 ± 0.25###

*Note*: There were 6 rats in each group. ***p < 0.001 vs. control group; ###*p* < 0.001 vs. model group.

### 3.4 Effects of Suanzao Ren Decoction on Histopathological Changes and Oxidative Stress in the Hippocampus of CUS Rats

**FIGURE 5:**
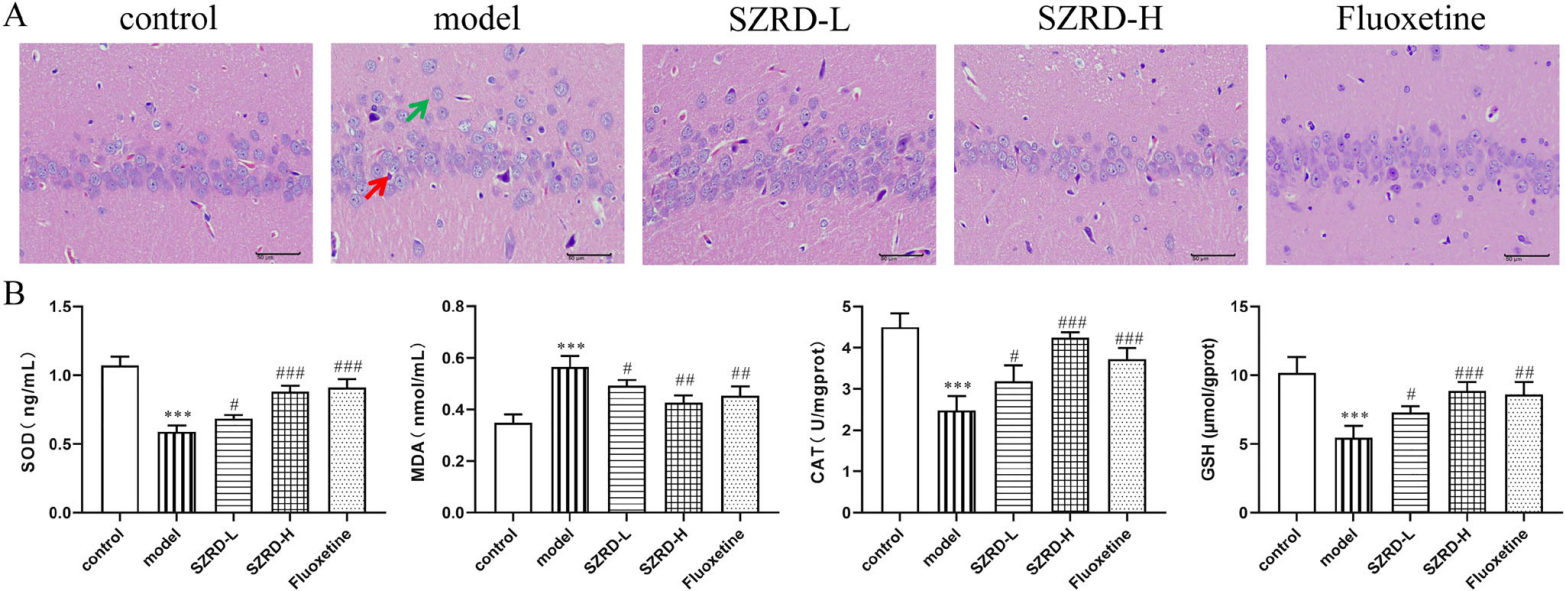
Effects of Suanzao Ren Decoction on histopathological changes and oxidative stress in the hippocampus of CUS rats. (A) HE staining of CA1 region of the hippocampus in CUS rats (×400, 50 µm). Red arrow: neuronal necrosis; blue arrow: neuronal arrangement is loose and disordered. (B) The levels of SOD, MDA, CAT, and GSH in the hippocampus were detected by ELISA. There were 6 rats in each group (SOD: model group vs. SZRD‐L group, *p =* 0.038. MDA: model group vs. SZRD‐L group, *p =* 0.041; model group vs. SZRD‐H group, *p =* 0.002; model group vs. fluoxetine group, *p =* 0.007. CAT: model group vs. SZRD‐L group, *p =* 0.019; model group vs. fluoxetine group, *p =* 0.001. GSH: model group vs. SZRD‐L group, *p =* 0.021; model group vs. SZRD‐H group, *p =* 0.001; model group vs. fluoxetine group, *p =* 0.001). *p < 0.001 vs. control group; #*p* < 0.05, ##*p* < 0.01, ###*p* < 0.001 vs. model group.

The HE staining results (Figure 5A) showed that in the control group, pyramidal cells in the CA1 hippocampal region exhibited dense arrangement with regular morphology, large and round nuclei, and abundant cytoplasm. In the model group, pyramidal cells in the CA1 area were significantly reduced, characterized by enlarged intercellular spaces, disordered arrangement, and a small number of necrotic cells with shrunken nuclei. After treatment with Suanzao Ren Decoction, these pathological abnormalities were markedly improved. Oxidative stress is recognized as a key factor in anxiety disorders (Fu et al. 2023). Our study investigated whether Suanzao Ren Decoction could enhance antioxidant capacity to alleviate anxiety. We investigated whether Suanzao Ren Decoction could regulate antioxidant capacity and improve anxiety. SOD is an important component of the antioxidant enzyme system in biological systems. MDA is one of the main products produced during lipid peroxidation. The ELISA results (Figure 5B) showed that compared with the control group, the SOD, CAT, and GSH levels in the model group were significantly reduced, and the MDA level was significantly increased; when treated with different concentrations of Suanzao Ren Decoction, the SOD, CAT, and GSH levels were significantly increased, and the MDA level was significantly decreased.

### 3.5 Effects of Suanzao Ren Decoction on Cognitive Dysfunction in Hippocampal Tissue of CUS Rats

**FIGURE 6:**
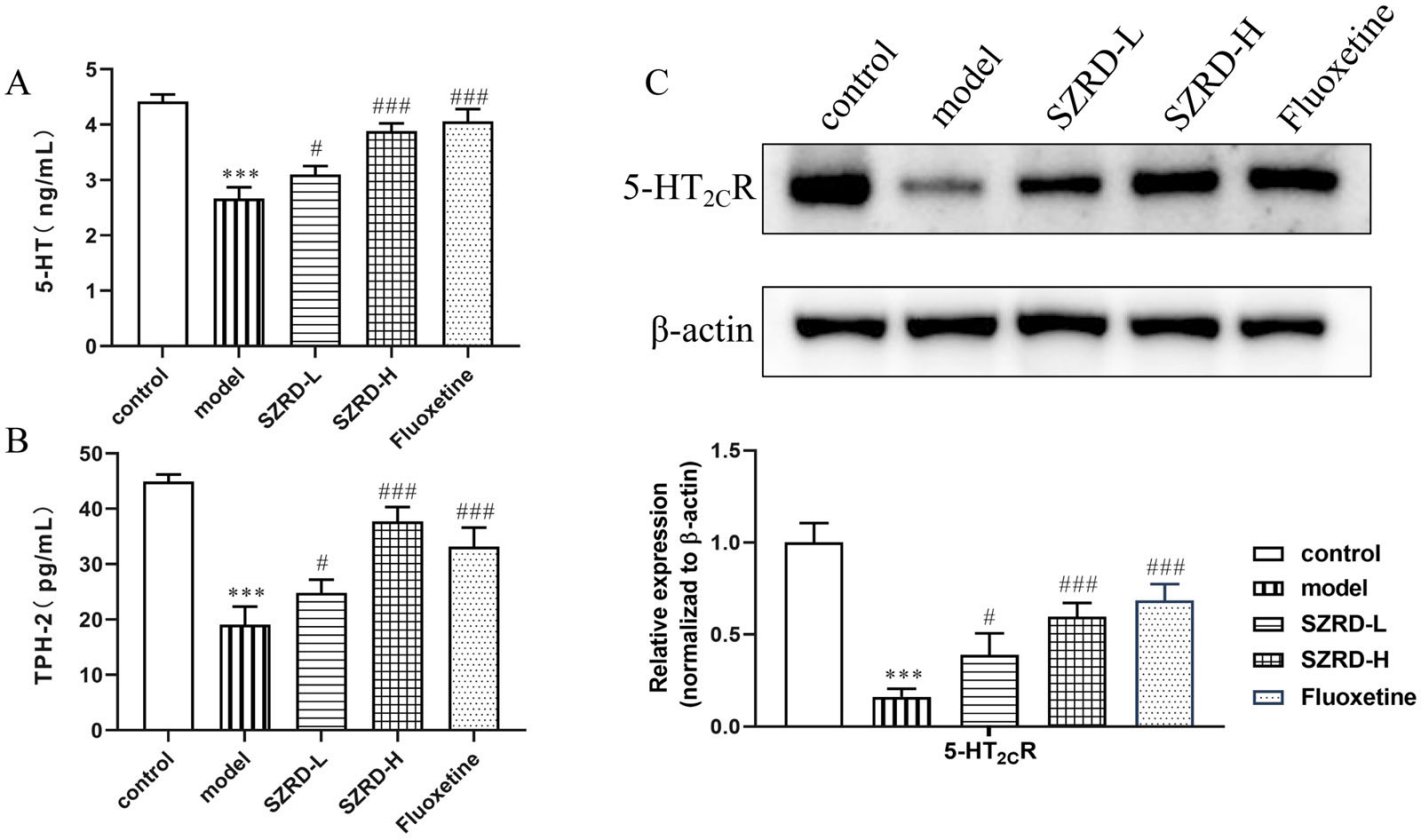
**Effects of Suanzao Ren Decoction on cognitive dysfunction in hippocampal tissue of CUS rats**. (A) ELISA was used to detect 5‐HT levels in hippocampal tissue. (B) ELISA was used to detect TPH‐2 levels in hippocampal tissue. (C) Western blotting was used to detect the expression of 5‐HT_2C_R protein in hippocampal tissue. There were 6 rats in each group (5‐HT: model group vs. SZRD‐L group, *p =* 0.043. TPH‐2: model group vs. SZRD‐L group, *p* = 0.047. 5‐HT_2C_R: model group vs. SZRD‐L group, *p =* 0.01). *p < 0.001 vs. vs. control group; #*p* < 0.05, ###*p* < 0.001 vs. model group.

Currently, the most common treatment drugs are selective 5‐HT reuptake inhibitors (fluoxetine) (Oberste et al. 2020), which can improve anxiety symptoms by regulating the 5‐HT neurotransmitter system. 5‐HT is closely related to mood regulation, and its dysfunction is significantly associated with the occurrence and development of anxiety disorders. TPH‐2 is a key enzyme in 5‐HT synthesis in the central nervous system, and its activity and expression level directly affect 5‐HT synthesis. In clinical studies, the genetic polymorphism of TPH‐2 is associated with the efficacy of antidepressants, suggesting that it may be of great significance in the treatment of anxiety disorders. The results showed that compared with the control group, the levels of 5‐HT, TPH‐2, and 5‐HT_2C_R in the model group were significantly reduced. After treatment with Suanzao Ren Decoction, the levels of 5‐HT, TPH‐2, and 5‐HT_2C_R were significantly increased (Figure 6A, B).

### 3.6 Effects of Suanzao Ren Decoction on Autophagy in Hippocampal Tissue of CUS Rats

**FIGURE 7:**
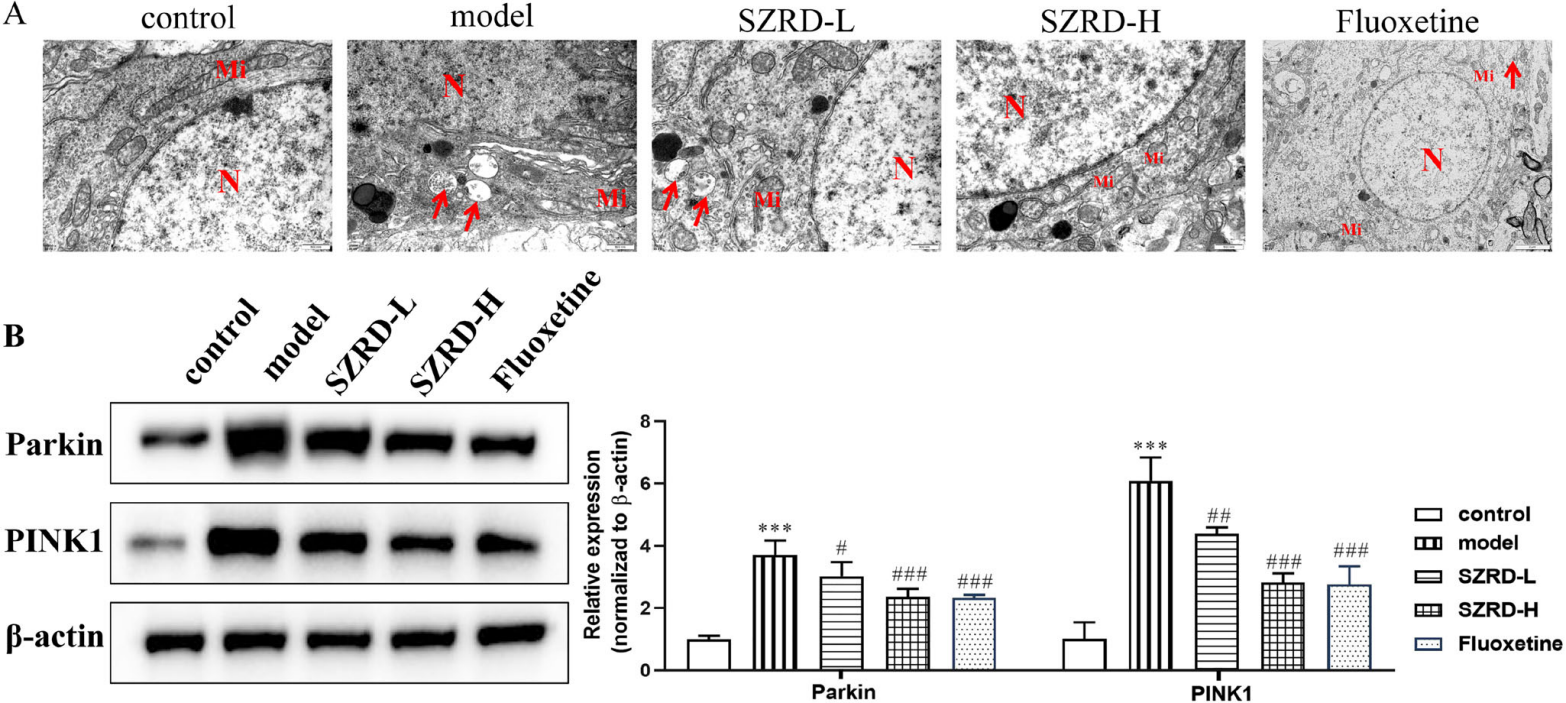
**Effects of Suanzao Ren Decoction on autophagy in hippocampal tissue of CUS rats**. (A) Transmission electron microscopy observation of mitochondrial autophagy in hippocampal tissue. Magnification: ×25,000 times. N: nucleus, Mi: mitochondria, red arrows: autophagolysosomes. (B) Western blotting detection of Parkin and PINK1 protein expression in hippocampal tissue. There were 6 rats in each group (Parkin: model group vs. SZRD‐L group, *p =* 0.024; model group vs. SZRD‐H group, *p =* 0.001. PINK1: model group vs. SZRD‐L group, *p =* 0.003). ^***^ *p* < 0.001 vs. control group; ^#^ *p* < 0.05, ^##^ *p* < 0.01, ^###^ *p* < 0.001 vs. model group.

Oxidative stress is related to the intracellular mitochondria. When oxidative stress occurs, the generation of ROS in mitochondria increases significantly, leading to mitochondrial dysfunction and activation of a series of signaling pathways (such as the PINK1/Parkin pathway), thus inducing mitochondrial autophagy. TEM results (Figure 7A) demonstrated that neuronal morphology in the control group was relatively normal. The mitochondria exhibited an oval or rod‐like shape, with clearly discernible cristae that were either lamellar or tubular in structure. In contrast, neurons in the model group displayed shrunken and irregularly shaped nuclei, swollen mitochondria, reduced, fragmented, or completely absent cristae. Additionally, a significant number of autophagic lysosomes were observed within the cytoplasm in the model group. Suanzao Ren Decoction reduced mitochondrial damage and inhibited autophagy. Western blotting results (Figure 7B) showed that the expression of Parkin and PINK1 in the model group was higher than that in the control group, and the treatment with Suanzao Ren Decoction decreased the expression of Parkin and PINK1. This suggested that anxiety leads to excessive autophagy of mitochondria in the hippocampus, and Suanzao Ren Decoction can relieve excessive autophagy of mitochondria in anxiety.

## 4 Discussion and Conclusion

Anxiety disorder is a common mental disorder characterized by excessive and persistent worry, fear, and avoidance reactions, which are different from the transient anxiety and fear in daily life (Craske et al. 2017). It has become a mental and psychological public health issue that has attracted much attention. Therefore, the treatment of anxiety disorders has become a research hotspot in recent years. The exact pathogenesis of anxiety disorders remains to be elucidated. The possible causes currently identified include abnormalities in neurotransmitters, neuroendocrine disorders, changes in neuroplasticity, genetic factors, and psychosocial factors (Freeman 2022). Oxidative stress leading to mitochondrial dysfunction may be a pathogenic mechanism of anxiety disorder. Therefore, inhibiting oxidative stress and improving mitochondrial function may be a new strategy for the treatment of anxiety disorders. TCM herbs, with their characteristics of multiple components, multiple targets, and multiple pathways, can exert anti‐anxiety effects through mechanisms such as regulating neurotransmitters, anti‐oxidant stress, and anti‐inflammatory actions (Liu et al. 2025). At present, TCM herbs have become the first choice for anti‐anxiety drugs. Modern research has revealed the multi‐component, multi‐target pharmacological profile of Suanzao Ren Decoction. Ziziphi Spinosi Semen (Suan zao ren), the monarch herb, contains jujubosides and flavonoids that exert sedative‐hypnotic and anxiolytic effects via the GABAergic and serotonergic systems. Anemarrhenae Rhizoma (Zhi mu) exhibits antidepressant and neuroprotective properties through antioxidant and anti‐inflammatory pathways. Poria (Fu ling) has been reported to regulate monoamine neurotransmitters and alleviate anxiety‐like behaviors. Chuanxiong Rhizoma (Chuan xiong) improves cerebral microcirculation and protects against oxidative stress‐induced neuronal injury. Glycyrrhizae Radix (Gan cao) possesses anti‐inflammatory and harmonizing effects. Collectively, these findings suggest that Suanzao Ren Decoction may confer anti‐anxiety and cognitive‐protective benefits through coordinated regulation of neurotransmitter homeostasis and redox balance, providing a pharmacological rationale for the present investigation. The study constructed a CUS model to explore the possibility that Suanzao Ren Decoction may improve anxiety disorders by regulating neurotransmitters, inhibiting oxidative stress, and excessive mitochondrial autophagy.

The hippocampus is involved in the regulation of learning and memory, emotions, and behavior. Low mood can affect judgment, analytical skills, and cognitive abilities, which in turn impact the daily life, study, and work of patients with anxiety disorder (Wang et al. 2022). The hippocampus is damaged to varying degrees in various stress‐induced diseases and is considered an important component in the development of anxiety and depression (Zhang et al. 2022). It has been observed that the atrophy of hippocampal neurons indicates cellular dysfunction, which is consistent with the characteristics of clinical populations with anxiety and depression (Bjørklund et al. 2021). The study results showed that in CUS rats, the number of pyramidal cells in the CA1 region of the hippocampus is significantly reduced, with loose and disordered arrangement, and a small amount of necrosis. Treatment with Suanzao Ren Decoction at different concentrations significantly improved the pathological damage in the CA1 region of the hippocampus.

5‐HT plays an important role in anti‐anxiety, depression, and cognitive dysfunction (Gupta et al. 2021; Zhou et al. 2022). 5‐HT receptor agonists can significantly improve hippocampal nerve damage and thus reduce cognitive dysfunction (Ma et al. 2023). As a rate‐limiting enzyme of 5‐HT synthesis, TPH‐2 affects the central 5‐HT concentration (Kim et al. 2018). The high expression of 5‐HT_2C_R in the brain, especially in the prefrontal cortex, can participate in the regulation of the 5‐HT system. Studies have shown that activation of 5‐HT_2C_R can alleviate the cognitive impairment caused by chronic intermittent alcohol consumption (Flanigan et al. 2023). In this study, the levels of 5‐HT, TPH‐2, and 5‐HT_2C_R in CUS rats were significantly reduced. After treatment with different concentrations of Suanzao Ren Decoction, the levels of 5‐HT, TPH‐2, and 5‐HT_2C_R were significantly increased. This indicates that Suanzao Ren Decoction may improve anxiety‐like behavior and cognitive dysfunction by regulating the 5‐HT neurotransmitter system.

It is well known that learning and cognitive dysfunction are mainly related to oxidative stress injury, autophagy dysfunction, apoptosis and so on (Gupta and Ambasta 2021). Recent studies have shown that oxidative stress may be an important trigger for anxiety disorders. Some literature suggests that oxidative stress may lead to the development of anxiety disorders, and oxidative stress model mice showed significant anxiety‐like behavior, which could be antagonized by antioxidants (Yin et al. 2024). MDA is the final product of lipid oxidation, and its content can reflect the degree of cell damage (Liu et al. 2025). SOD is an important antioxidant in the body, and its activity can represent the strength of the body's antioxidant capacity (Gulcin 2025). Therefore, the level of MDA and SOD in vivo is usually used as the main index to detect oxidative stress (Bai et al. 2025). CAT is a key antioxidant enzyme that catalyzes the decomposition of hydrogen peroxide into water and oxygen, thereby preventing oxidative damage to cells. GSH, as a major non‐enzymatic antioxidant, directly scavenges reactive oxygen species and maintains cellular redox homeostasis. This study also confirmed that CUS rats showed significantly reduced SOD, CAT, and GSH levels and significantly increased MDA level. After treatment with different concentrations of Suanzao Ren Decoction, SOD, CAT, and GSH levels were significantly increased, and MDA level was significantly decreased. This indicated that Suanzao Ren Decoction may inhibit intracellular oxidative stress and improve anxiety by regulating the expression of SOD, CAT, and GSH and MDA.

Disruption of mitochondrial autophagy is one of the pathogenic mechanisms of anxiety and depression. In this study, neurons in the hippocampal tissue of CUS rats were damaged, with mitochondrial swelling and an increased number of autophagolysosomes. It is speculated that the occurrence of anxiety‐like behavior in rats may be related to excessive activation of mitochondrial autophagy. PINK1/Parkin is one of the most important and recognized signaling pathways regulating mitochondrial autophagy. The relative mRNA expression level of PINK1, a mitochondrial autophagy gene, was decreased in the serum of patients with anxiety and depression (Gupta and Ambasta 2021). This study also confirmed a significant increase in Parkin and PINK1 protein expression in CUS rats. After administration of Suanzao Ren Decoction at different concentrations, the expression levels of these proteins were significantly reduced. This revealed that the mechanism by which Suanzao Ren Decoction improves mitochondrial autophagy is related to its regulation of the PINK1/Parkin signaling pathway.

In conclusion, this study demonstrated that CUS‐induced anxiety is associated with oxidative stress, excessive mitochondrial autophagy, and neurotransmitter dysregulation. Suanzao Ren Decoction alleviates anxiety‐like behavior and cognitive dysfunction in CUS rats by restoring neurotransmitter homeostasis, attenuating oxidative stress, and suppressing excessive mitochondrial autophagy. However, this study also has some limitations. First, although we observed significant alterations in PINK1/Parkin‐mediated mitochondrial autophagy and associated oxidative stress markers, these results remain correlational; definitive causal links between the inhibition of excessive mitochondrial autophagy and the amelioration of anxiety‐like behavior await further validation through rescue experiments employing autophagy‐specific modulators or genetic interventions. Second, although LC‐MS/MS profiling identified numerous constituents of Suanzao Ren decoction, the specific bioactive phytochemicals responsible for the observed anti‐anxiety and cognitive‐protective effects have not been isolated or functionally verified, warranting future studies integrating network pharmacology, molecular docking, and monomer‐based experiments to elucidate the precise material basis and targets of this classical formula.

## Author Contributions

Tianyan Luo: writing – original draft, writing – review and editing, validation, supervision, visualization. Guifang Xiang: conceptualization, methodology, software, data curation, investigation, formal analysis, funding acquisition, project administration, resources. Qing Liu: conceptualization, methodology, software, data curation, investigation, formal analysis, funding acquisition, project administration, resources.

## Conflicts of Interest

The authors declare that there is no conflict of interest regarding the publication of this paper.

## Funding

This work was supported by Sichuan Province Medical Research Project Plan (Grant No. 2022‐CXTD‐06).

## Data Availability

The datasets generated and or analyzed during the present study are available from the corresponding author.
